# Controle do Intervalo QT para Prevenção de Torsades de Pointes Durante uso de Hidroxicloroquina e/ou Azitromicina em Pacientes com COVID 19

**DOI:** 10.36660/abc.20200389

**Published:** 2020-06-29

**Authors:** Tan Chen Wu, Luciana Sacilotto, Francisco Carlos da Costa Darrieux, Cristiano Faria Pisani, Sissy Lara de Melo, Denise Tessariol Hachul, Mauricio Scanavacca

**Affiliations:** 1 Universidade de São Paulo Faculdade de Medicina Hospital das Clínicas São PauloSP Brasil Universidade de São Paulo Faculdade de Medicina Hospital das Clínicas Instituto do Coração – Arritmia, São Paulo, SP - Brasil

**Keywords:** Coronavirus/complicações, COVID-19, Pandemia, Torsades Pointes, Taquicardia Ventricular, Hidroxicloroquina/uso terapêutico, Azitromicina/uso terapêutico, Arritmias

## Introdução

Em dezembro de 2019, foram relatados os primeiros casos da doença causada pelo novo coronavírus (COVID-19), originários de Wuhan, China.^1^ Desde a declaração de pandemia em março de 2020 por Organização Mundial da Saúde (OMS), com disseminação intercontinental, vivenciamos intensa busca por um tratamento seguro e eficaz.

Estudos *in vitro* demonstraram algum efeito da cloroquina contra o novo coronavírus,^3^ intermediada pela glicosilação dos receptores celulares de SARS-CoV e pelo aumento do pH endossômico, bloqueando a invasão celular pelo vírus.^4^ Além dessa atividade antiviral, a cloroquina, tradicionalmente um imunomodulador, emergiu como promissora no tratamento da pneumonia que se instala em torno de uma semana após o início dos sintomas.^5^

A hidroxicloroquina (HCQ), derivada da cloroquina, tem efeitos terapêuticos semelhantes e menos efeitos adversos, sendo amplamente utilizada em doenças autoimunes. Os primeiros ensaios clínicos com a HCQ, no tratamento do COVID-19, reforçaram um aparente benefício e encorajaram a sua aprovação para estudos clínicos por órgãos regulatórios nacionais e internacionais.^6 - 8^

O macrolídeo azitromicina (AZ), ainda por mecanismo incerto, demonstrou ser efetivo quando iniciado precocemente em pacientes com infecções respiratórias graves.^9^ Embora estas medicações tenham um adequado perfil de segurança em diversas situações clínicas, ambos bloqueiam o canal de potássio hERG, podendo prolongar a repolarização ventricular e causar *torsades de pointes* (TdP);^10 - 11^

O subgrupo da população com maior risco de eventos potencialmente fatais são os pacientes com múltiplas comorbidades ou em cuidados intensivos, que estarão expostos a interações medicamentosas e/ou a distúrbios eletrolíticos, além dos portadores da síndrome do QT longo congênito, que podem necessitar do tratamento (1:2000 indivíduos).^12^ A avaliação do risco antes e o monitoramento do intervalo QTc durante são fundamentais para prevenção de eventos arrítmicos.

Giudicessi et al.,^13^ divulgaram uma diretriz institucional da *Mayo Clinic* , para a segurança dos pacientes em uso de HCQ e/ou AZ.^13^ O *American College of Cardiology* apresentou uma sugestão para controle do intervalo QT e prevenção de arritmias ventriculares em pacientes que participam do protocolo HQC/AZ para tratamento do COVID-19.^14^ 0 Núcleo de Arritmias do Instituto do Coração formulou um protocolo institucional a fim de contribuir para o uso consciente dessas medicações durante o surto de infeção por COVID-19.

## Definição

**Figura 1:**
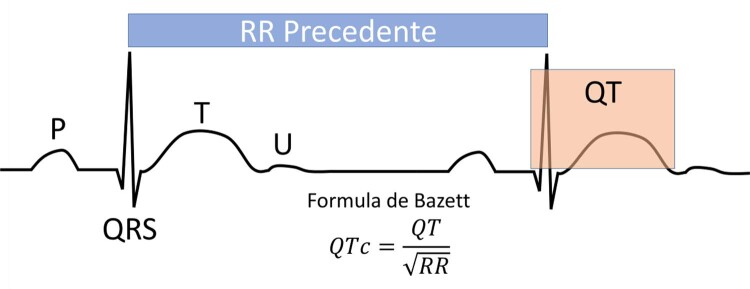
– *Intervalo QT. Fonte: Acervo InCor HCFMUSP*

**Figura 2:**
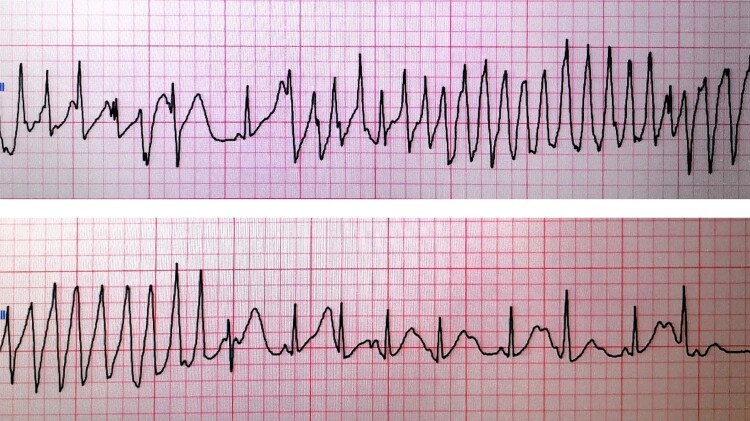
– *QT longo com torsades de pointes, Fonte: Acervo InCor HCFMUSP*

O intervalo QT é a medida da duração do início do complexo QRS até o final da onda T e é modulado pela frequência cardíaca ( Figura 1 ). Quando prolongado, está associado a maior risco de ocorrência de arritmias ventriculares polimórficas e TdP ( Figura 2 ).^15^ A medida do intervalo QT deve ser corrigida pela frequência cardíaca (QTc) e, na população adulta, é considerada normal quando ≤ 440 ms em homens e ≤ 460 ms em mulheres.^16^

## Como Medir o Intervalo QTc

**Figura 3:**
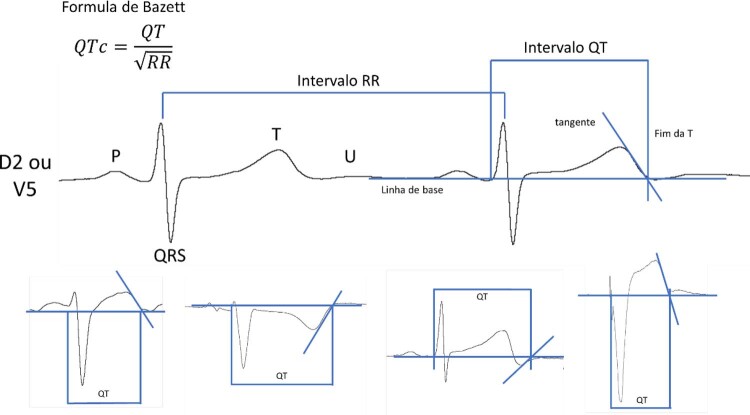
– *Exemplos de determinação do intervalo QT pelo método de tangente. Fonte: Acervo InCor HCFMUSP,*

O intervalo QT pode ser medido pelo método de tangente ( Figura 3 ) ou visual (quando o final da onda T for de fácil definição), preferencialmente em derivações DII ou V5. ^17^

A correção pela frequência cardíaca pode ser feita pela fórmula de Bazett, considerando-se o RR precedente ao intervalo QT medido (QTc= intervalo QT/raiz quadrada do intervalo RR). Essa fórmula está disponível em calculadoras de *sites* (QTc calculadora) ou em Apps (por exemplo: EP Mobile ou MedCalX).

## Monitoramento do intervalo QTc durante o tratamento com HCQ/AZ

Após avaliação do ECG inicial, os pacientes podem ser estratificados conforme o risco de desenvolver TdP, em menor risco (grupo verde), risco intermediário (grupo azul), risco intermediário a elevado (grupo laranja) e alto risco (grupo vermelho).

**Figura 4:**
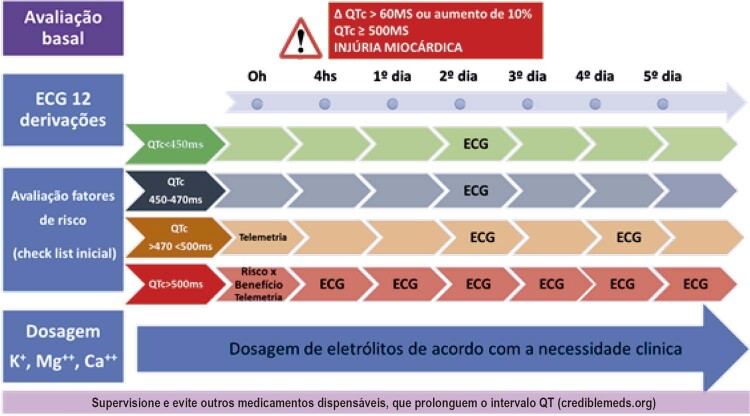
– *Esquema sugerido de controle do tratamento HCQ e/ou AZ*

A monitorização após início do tratamento pode ser feita pelo ECG convencional de 12 derivações, ECG apenas com derivações periféricas, pela telemetria ou por outros dispositivos remotos, para nessa situação peculiar de pandemia, minimizar a exposição de profissionais de saúde e de equipamentos ao vírus. Sugerimos que a frequência de monitorização eletrocardiográfica e o método (ECG, telemetria ou dispositivos) sejam determinados pelo risco do paciente, baseado em um QTc inicial (admissional). A figura 4 esquematiza o modelo de controle proposto.

### Quanto à avaliação de risco inicial do paciente para o tratamento pela medida do QT basal em ECG de 12 derivações:

**Table:** 

QTc 450 ms	Liberado para uso
450ms < QTc ≤ 470 ms	Cautela ou apenas uso em regime hospitalar
470ms < QTc < 500 ms	Evitar ou somente uso hospitalar com telemetria
QTc 500 ms	Evitar, considerando o risco/benefício

Em casos de dúvidas ou medições limítrofes para maior risco ao longo do tratamento, pode-se optar pelo uso isolado da HCQ ou da AZ ou também pelo uso escalonado da HCQ, seguido da AZ, sob monitorização. Sugere-se decisão compartilhada com a equipe de cardiologia ou arritmia do hospital.

### Quando repetir o ECG durante o tratamento hospitalar de acordo com o QTc prévio

**Table:** 

QTc 450 ms	No 2º dia
450 ms < QTc ≤ 470 ms	No 2º dia
470 ms < QTc < 500 ms	No 2º dia e no 4º dia
QTc 500 ms	Em 4 a 8 horas após a primeira dose e diariamente

### Intensificar o controle nas seguintes condições:

- Se houver fatores de risco associados ( Tabela 1 ).
  **Tabela 1:** – Fatores de risco para prolongamento de QT e TdP. (18)

Idade > 65 anos Mulheres Distúrbios eletrolíticos (hipocalcemia, hipocalemia, hipomagnesemia) Uso concomitante de mais medicações que prolongam QT (crediblemeds.org) Insuficiência coronariana aguda Insuficiencia cardiaca crônica ou FEVE < 40% Bradicardia, bloqueio de ramo Cardiomiopatia hipertrófica Síndrome do QT longo congênito ou outra susceptibilidade genética Diabetes mellitus Insuficiência renal crônica dialítica Anorexia ou inanição Hipoglicemia Feocromocitoma Pós-parada cardiorrespiratória recente Pós-hemorragia subaracnóidea, acidente vascular cerebral ou traumatismo crânio encefálico (1º semana).
- Na presença de complicações cardiovasculares como miocardite e isquemia miocárdica.

**Figura 5:**
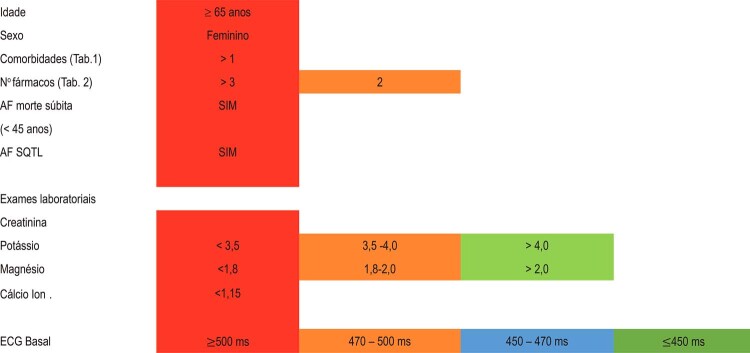
– *Lista de checagem PRÉ-tratamento. AF= antecedente familiar; SQTL = síndrome do QT longo congênito* Em vermelho: atenção às condições de risco; em laranja: moderado risco; em verde: baixo risco ou alvo desejável

Obs.: Sugestão de modelo para Lista de checagem pré-tratameto e controle apresentadas nas Figuras 5 e 6 .

**Figura 6:**
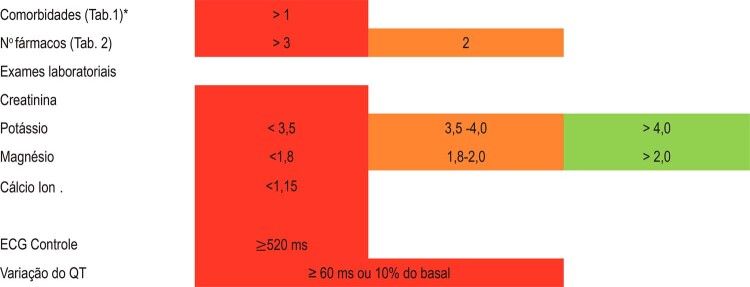
– *Lista de checagem CONTROLE:* * Condições clínicas e metabólicas durante a evolução clínica: injurias miocárdicas entre outras.

### Sinais de alerta

- Aumento do QTc > 60 ms e/ou mais de 10% em relação ao ECG basal.
- QTc acima de 520 ms - avaliar suspensão do tratamento depois de serem suspensos outros fármacos (dispensáveis e com efeito sinérgico sobre o QTc) ou distúrbio eletrolítico.
- Necessidade de adicionar medicações que prolongam o intervalo QT, conforme evolução clínica do paciente.
- Presença de arritmia ventricular e/ou bradicardia associada -> escolher os fármacos que podem ser suspensos conforme o balanço risco *vs* . benefício. Nessas situações, há necessidade de manter o paciente sob telemetria contínua.

## Cuidados adicionais para prevenção de TdP

### Quanto ao controle de eletrólitos na admissão:

A dosagem de cálcio, potássio e magnésio, fundamentais na estabilidade da repolarização ventricular, deve ser realizada para todos os pacientes elegíveis para o tratamento com HCQ/AZ.

- Manter K^+^ > 4,0
- Manter Mg^++^ > 2,0
- Evitar hipocalcemia

Obs.: mesmo em pacientes com nível sanguíneo normal, recomenda-se manter suplementação empírica de magnésio via oral, exceto naqueles com insuficiência renal (ClCr < 30 ml/min).

### Quanto ao controle de eletrólitos na evolução:

A rotina de monitorização de eletrólitos deve ser realizada a critério clínico, sempre que houver necessidade de ajustes para manter os níveis ideais ou desejáveis durante o tratamento, principalmente nos pacientes com intervalo QTc inicial > 470 ms.

### Quanto ao uso de medicações concomitantes:

Deve-se evitar a prescrição de outros medicamentos, não essenciais, que prolonguem o intervalo QT. Inúmeras drogas, utilizadas habitualmente em pacientes internados, podem bloquear o canal hERG, prolongar o tempo de repolarização ventricular e facilitar a ocorrência de TdP.^18^ A supervisão de farmacêuticos é desejável sempre que possível, para garantir a segurança do paciente.

Na Tabela 2 elencamos as medicações de risco baixo (verde), risco possível (laranja) e alto risco (vermelho) de prolongamento do intervalo QT e ocorrência de TdP. Assim, sempre que possível, deve-se preferir as medicações adicionais de baixo risco, pois tanto a HCQ como a AZ já são listadas como de alto risco para ocorrência de TdP.

**Tabela 2:** – Lista de medicações a serem evitadas (em vermelho e laranja)

	Alto risco	Moderado risco	Baixo risco ou NC
Antiarrítmicos	Amiodarona Sotalol	Propafenona	Lidocaína Propranolol Sulfato Mg Isoproterenol
Antipsicóticos	Haloperidol	Risperidona	Benzodiazepínico
	Clorpromazina	Quetiapina	
	Levomepromazine	Prometazina Olanzapina	
Sedativos	Propofol	Dexmedetomidina	Midazolam
			Fentanil
Antieméticos e pró-cinéticos	Ondansentrona Domperidona Bromoprida Cisaprida	Cimetidina Granisetrona Metoclopramida	Dimenidrato
Antibióticos	Quinolonas	Piperacilina-tazobactam Sulfametoxazole-trimetropim	Teicoplamida Vancomicina
Antifúngicos	Fluconazol	Anfotericina Itraconazol Voriconazol	
Inibidores de bomba de prótons		Pantoprazol	
		Omeprazol Esomeprazol Lanzoprazol	
Antialérgicos		Prometazina	Fexofenadina
		Hidroxizina Difenidramina	Loratadina
Pandemia	Cloroquina Azitromicina		Oseltamivir
Broncodilatadores		Salbutamol Fenoterol Formoterol Terbutalina	
Anticolinesterázicos	Donepezila	Galantamina	
Antidepressivos	Citalopram Escitalopram	Fluoxetina Paroxetina Mirtazapina Tricíclicos Sertralina Venlafaxina	
Outros	Cilostazol Metadona Tramadol	Loperamida	Fenitoína
Cuidados especiais			
Diuréticos	Cuidados com espoliação de eletrólitos		

*NC – Não classificada, ou seja, sem evidência de prolongar o intervalo QT com base nos estudos publicados.*

Alguns medicamentos podem aumentar o risco por outros mecanismos ou de maneira indireta, como no caso da hipocalemia induzida por diuréticos. A lista completa de interações medicamentosa deve ser checada diariamente pelo site crediblemeds.org.^19^

### Em caso de ocorrência de arritmia ventricular ou TdP (Tabela 3) 20,21:

- Lidocaína é o antiarrítmico de escolha:
- Sulfato de magnésio
- Isoprotenerol em TdP mediado por bradicardia
- Marcapasso provisório para pacientes bradicárdicos, com TdP recorrente. A frequência cardíaca inicial deve ser programada para 90 bpm e os ajustes feitos de acordo com a resposta clinica do paciente.
- Suspensão imediata do uso de todas as medicações com potencial para prolongamento do intervalo QT.

## Conclusão

O risco de arritmias fatais, facilitado pelo uso da HCQ e/ou AZ, em pacientes com infecção por COVID-19, ou em outras situações diárias fora da pandemia com medicações que tenham potencial para prolongamento do intervalo QT, pode ser minimizado com a aplicação de protocolos de conduta que auxiliem o profissional de saúde na decisão pela prescrição e manutenção do tratamento.

**Tabela 3:** – Manejo Farmacológico da arritmia ventricular e/ou TdP

**Lidocaína**
Dose de ataque – 1,0 a 1,5mg/kg IV com doses repetidas em *bolus* de 0,5-0,75 mg/kg em *bolus* até 3 mg/kg. Manutenção – 20 mcg/kg/min IV.
**Sulfato de magnésio**
2 a 4 g IV
**Isoprotenerol**
Dose de ataque: 1 a 2 mcg IV. Manutenção; 0,15 mcg/min e titular até 0,3 mcg/min de acordo com a resposta ou necessidade clínica.
