# Supplementary material for: Controle do Intervalo QT para Prevenção de Torsades de Pointes Durante uso de Hidroxicloroquina e/ou Azitromicina em Pacientes com COVID 19
Source: Arq Bras Cardiol. 2020 Jun 29;114(6):1061–6. [Article in Portuguese] doi: 10.36660/abc.20200389 (PMC8416123; doi:10.36660/abc.20200389)
Supplement: Translation

# Translation: QT Interval Control to Prevent Torsades de Pointes during Use of Hydroxychloroquine and/or Azithromycin in Patients with COVID-19

**Authors:** Tan Chen Wu, Luciana Sacilotto, Francisco Carlos da Costa Darrieux, Cristiano Faria Pisani, Sissy Lara de Melo, Denise Tessariol Hachul, Mauricio Scanavacca

**Affiliations:** 1 Brazil Universidade de São Paulo Faculdade de Medicina Hospital das Clínicas Instituto do Coração – Arritmia, São Paulo, SP - Brazil

**Keywords:** Coronavirus/complications, COVID-19, Pandemics, Torsades Pointes, Tachycardia,Ventricular, Hydroxychloroquine/therapeutic, use, Arrhythmias

## Introduction

In December 2019, the first cases of the novel coronavirus disease (COVID-19) were reported in Wuhan, China.^1^ Since the pandemic designation in March 2020 by the World Health Organization (WHO), with intercontinental spread of the disease, we are intensely seeking for a safe and effective treatment.^2^

*In vitro* studies have demonstrated some effect of chloroquine against the new coronavirus,^3^ mediated by the glycosylation of SARS-CoV cell receptors and by increased endosomal pH, blocking cell invasion by the virus.^4^ In addition to this antiviral activity, chloroquine, which is traditionally an immunomodulator, has shown to be promising for treatment of pneumonia that installs approximately one week after onset of symptoms.^5^

Hydroxychloroquine (HCQ), which is derived from chloroquine, has similar therapeutic effects, with fewer adverse effects, and it is widely used in autoimmune diseases. The first clinical trials with HCQ for treatment of COVID-19 reinforced an apparent benefit and encouraged its approval for clinical studies by national and international regulatory institutions.^6 - 8^

The macrolide azithromycin (AZ), due to a mechanism that is still unclear, has shown to be effective when initiated early in patients with severe respiratory infections.^9^ Although these medications have an adequate safety profile in diverse clinical situations, both of them block the hERG potassium channel, which can prolong ventricular repolarization and cause torsades de pointes (TdP).^10 , 11^

The subgroup of the population with the highest risk of potentially fatal events are patients with multiple comorbidities or patients in intensive care, who will be exposed to drug interactions and/or electrolyte disorders, in addition to patients with congenital long QT syndrome, who may need treatment (1:2000 individuals).^12^ Risk assessment before treatment and monitoring of the QTc interval during treatment are essential measures to preventing arrhythmic events.

Giudicessi et al.^13^ published an institutional guideline from the Mayo Clinic for safety of patients receiving HCQ and/or AZ.^13^ The American College of Cardiology suggested controlling the QT interval and preventing ventricular arrhythmias in patients participating in the HQC/AZ protocol for treating COVID-19.^14^ The Arrhythmia Center of the Heart Institute of the University of São Paulo formulated an institutional protocol in order to contribute to the conscious use of these medications during the COVID-19 outbreak.

## Definition

The QT interval is the measurement of the duration from the beginning of the QRS complex to the end of the T wave, which is modulated by heart rate ( Figure 1 ). When the interval is prolonged, it is associated with a greater risk of polymorphic ventricular arrhythmias and TdP ( Figure 2 ).^15^ Measurement of the QT interval should be corrected by heart rate (QTc); in the adult population, ≤ 440 ms is considered normal in men, and ≤ 460 ms is considered normal in women.^16^

**Figure 1:** – *QT Interval Source: Collection, Heart Institute of the University of São Paulo (InCor HCFMUSP).*

**Figure 2:** – *Long QT with torsades de pointes. Source: Collection, Heart Institute of the University of São Paulo (InCor HCFMUSP).*

### How to Measure the QTc Interval

The QT interval can be measured either by the tangent method ( Figure 3 ) or visually (when the end of the T wave is easy to define), preferably in leads DII or V5.^17^

**Figure 3:** – *Examples of measurement of QT interval by the tangent method. Source: Collection, Heart Institute of the University of São Paulo (InCor HCFMUSP).*

Heart rate correction can be done using Bazett’s formula, considering the RR interval preceding the measured QT interval (QTc = QT interval / square root of the RR interval). This formula is available on website calculators (QTc calculator) or in applications (for example, EP Mobile or MedCalX).

### Monitoring the QTc interval during treatment with HCQ/AZ

After evaluation of initial ECG, patients may be stratified by risk of developing TdP in the following manner: lower risk (green group), intermediate risk (blue group), intermediate to high risk (orange group), and high risk (red group).

Monitoring after the start of treatment can be done by conventional 12-lead ECG, ECG with limb leads only, telemetry, or other remote devices in order to minimize the exposure of health professionals and equipment to the virus during this particular pandemic situation. We recommend that the frequency of electrocardiographic monitoring and the method (ECG, telemetry, or devices) be determined according to patients’ risks, based on an initial QTc (upon admission to the hospital). Figure 4 outlines the proposed control model.

**Figure 4:** – *Suggested HCQ and / or AZ treatment control scheme.*

### Initial risk assessment for treatment according to baseline QT measurement on 12-lead ECG:

**Table:** 

QTc ≤ 450 ms	Approved for use
450ms < QTc ≤ 470 ms	Use with caution or only in the hospital
470ms < QTc < 500 ms	Avoid or only use in the hospital with telemetry
QTc ≥ 500 ms	Avoid, considering risk/benefit

In cases where doubts exist or in borderline measurements regarding greater risk throughout the treatment, it is possible to opt for isolated use of HCQ or AZ, or also for staggered use of HCQ, followed by AZ, under monitoring. It is recommended that a shared decision be reached with the hospital’s cardiology or arrhythmia team.

### When to repeat ECG during treatment in the hospital, according to previous QTc

**Table:** 

QTc ≤ 450 ms	On day 2
450 ms < QTc ≤ 470 ms	On day 2
470 ms < QTc < 500 ms	On days 2 and 4
QTc ≥ 500 ms	4 to 8 hours after the first dose, then daily

### Control should be intensified in the following conditions:

- If there are associated risk factors ( Table 1 ).
  **Table 1:** – Risk factors for prolonged QT and TdP. (18)

Age > 65 years Women Electrolyte disorders (hypocalcemia, hypokalemia, hypomagnesemia) Concomitant use of other medications that prolong QT (crediblemeds.org) Acute coronary failure Chronic heart failure or LVEF < 40% Bradycardia, branch block Hypertrophic cardiomyopathy Congenital long QT syndrome or other genetic susceptibility Diabetes mellitus Chronic renal failure on dialysis Anorexia or starvation Hypoglycemia Pheochromocytoma Recent post-cardiorespiratory arrest Post-subarachnoid hemorrhage, stroke, or traumatic brain injury (week 1).
- In the presence of cardiovascular complications, such as myocarditis and myocardial ischemia.

N.B.: Figures 5 and 6 show suggested models for pre-treatment and control checklists.

**Figure 5:** – *Pre-treatment Checklist: FH = family history; LQTS=long QT syndrome* Red: special attention to conditions of risk; orange: moderate risk; green: low risk or desirable target

**Figure 6:** – *Control Checklist.* * Clinical and metabolic conditions during clinical evolution: myocardial injuries, among others.

### Warning signs

- Increase in QTc by > 60 ms and/or by more than 10% with respect to baseline ECG.
- QTc above 520 ms: evaluate suspending treatment after other drugs (those that are dispensable and that have a synergistic effect on QTc) have been suspended, or electrolyte disturbance.
- Need to add medications that prolong the QT interval, according to the patient’s clinical evolution.
- Presence of ventricular arrhythmias and/or associated bradycardia -> Choose the drugs that can be suspended according to the risk-benefit ratio. In these situations, it is necessary to keep the patient on continuous telemetry.

## Additional care measures for preventing TdP

### Regarding electrolyte control upon hospital admission:

Measurements of calcium, potassium, and magnesium, which are essential for the stability of ventricular repolarization, should be carried out for all patients eligible for treatment with HCQ/AZ.

- Maintain K^+^> 4.0
- Maintain Mg^++^> 2.0
- Avoid hypocalcemia

N.B.: Even in patients with normal blood level, it is recommended to maintain empirical magnesium supplementation orally, except in those with renal failure (ClCr < 30 ml/min).

### Regarding electrolyte control during patient progression:

Electrolyte monitoring routine should be determined at clinical discretion, whenever adjustments are needed to maintain ideal or desirable levels during treatment, especially in patients with an initial QTc interval > 470ms.

### Regarding use of concomitant medications:

It is necessary to avoid prescribing other non-essential drugs that prolong the QT interval. Numerous drugs that are commonly used in hospitalized patients can block the hERG channel, prolong ventricular repolarization time, and facilitate the occurrence of TdP.^18^ It is important to supervise use of medication whenever possible in order to guarantee patient safety.

Table 2 provides lists of low risk (green), possible risk (orange), and high risk (red) medications with respect to prolongation of the QT interval and occurrence of TdP. Therefore, whenever possible, additional low-risk medications should be preferred, as both HCQ and AZ are already listed as high risk for the occurrence of TdP.

**Table 2:** – List of medications to avoid (red and orange)

	High risk	Moderate risk	Low risk or NC
Antiarrhythmic drugs	Amiodarone Sotalol	Propafenone	Lidocaine Propranolol Magnesium sulfate Isoproterenol
Antipsychotic drugs	Haloperidol	Risperidone	Benzodiazepine
	Chlorpromazine	Quetiapine	
	Levomepromazine	Promethazine Olanzapine	
Sedatives	Propofol	Dexmedetomidine	Midazolam
			Fentanyl
Antiemetic and prokinetic drugs	Ondansentron Domperidone Bromopride Cisapride	Cimetidine Granisetrone Metoclopramide	Dimenhydrinate
Antibiotics	Quinolones	Piperacillin/tazobactam Sulfamethoxazole/ trimethoprim	Teicoplanin Vancomycin
Antifungal drugs	Fluconazol	Anfotericina Itraconazol Voriconazol	
Proton pump inhibitors		Pantoprazol	
		Omeprazol Esomeprazol Lanzoprazol	
Antiallergic drugs		Promethazine	Fexofenadine
		Hydroxyzine Diphenhydramine	Loratadine
Pandemic	Chloroquine Azithromycin		Oseltamivir
Bronchodilators		Salbutamol Fenoterol Formoterol Terbutalina	
Anticholinesterase drugs	Donepezil	Galantamine	
Antidepressives	Citalopram Escitalopram	Fluoxetine Paroxetine Mirtazapine Tricyclics Sertraline Venlafaxine	
Others	Cilostazol Methadone Tramadol	Loperamide	Phenytoin
Special precautions			
Diuretics	Precaution with spoliation of electrolytes		

*NC – Not classified, i.e., absence of evidence of prolonged QT interval based on published studies.*

Some medications can increase risk through other mechanisms or indirectly, as is the case of hypokalemia induced by diuretics. The complete list of drug interactions should be checked daily by the website crediblemeds.org.^19^

### In the event of ventricular arrhythmia or TdP (Table 3): 20,21

- Lidocaine is the antiarrhythmic drug of choice:
- Magnesium sulfate
- Isoprotenerol for TdP mediated by bradycardia
- Provisional pacemaker for bradycardic patients with recurrent TdP. Initial heart rate should be programmed to 90 bpm and adjustments should be made according to patient’s clinical response.
- Immediately suspend the use of all medications with potential to prolong the QT interval.

## Conclusion

The risk of fatal arrhythmias, increased with the use of HCQ and/or AZ, in patients with COVID-19, or in other daily situations, outside the pandemic, with medications that may potentially prolong the QT interval, can be minimized with the application of conduct protocols that help healthcare professionals decide on prescription and maintenance of treatment.

**Table 3:** – Pharmacological management of ventricular arrhythmia and/or TdP

**Lidocaine**
Loading dose: 1.0 to 1.5 mg/kg IV with repeated doses in bolus, 0.5 – 0.75 mg/kg in a bolus up to 3 mg/kg. Maintenance dose: 20 mcg/kg/min IV.
**Magnesium sulfate**
2 to 4 g IV
**Isoprotenerol**
Loading dose: 1 to 2 mcg IV. Maintenance dose: 0.15 mcg/min and titer up to 0.3 mcg/min according to clinical response or necessity.
